# Large-cell neuroendocrine carcinoma of the gynecologic tract: Prevalence, survival outcomes, and associated factors

**DOI:** 10.3389/fonc.2022.970985

**Published:** 2022-11-15

**Authors:** Li Pang, Jie Chen, Xiaohan Chang

**Affiliations:** ^1^ Department of Obstetrics and Gynecology, Shengjing Hospital of China Medical University, Shenyang, Liaoning, China; ^2^ Centre of Journals, China Medical University, Shenyang, Liaoning, China

**Keywords:** Large-cell neuroendocrine carcinoma, SEER, ovarian, endometrial, cervical, prognostic factors

## Abstract

**Background:**

We aimed to assess the clinical behavior of gynecologic large-cell neuroendocrine carcinoma (LCNEC) *via* a retrospective analysis of data from 469 patients.

**Methods:**

Patients diagnosed with gynecologic LCNEC from 1988 to 2015 were identified using the Surveillance, Epidemiology, and End Results database. Univariate and multivariate Cox hazard regression analyses were performed to assess independent predictors of overall survival (OS) and cancer-specific survival (CSS). OS and CSS were also evaluated using the Kaplan–Meier method, and the effects of different treatment regimens on prognosis were compared according to disease stage.

**Results:**

Cervical, ovarian, and endometrial LCNEC were observed in 169, 219, and 79 patients, respectively. The 5-year OS rates for patients with cervical, ovarian, and endometrial LCNEC were 35.98%, 17.84%, and 23.21%, respectively, and the median duration of overall survival was 26, 11, and 11 months in each group. The 5-year CSS rates for the three groups were 45.23%, 19.23%, and 31.39%, respectively, and the median duration of CSS was 41, 12, and 11 months in each group. Multivariate analysis revealed that American Joint Committee on Cancer stage, lymph node metastasis, and chemotherapy were independent prognostic factors for OS and CSS in patients with cervical LCNEC. Lymph node metastasis, surgery, and chemotherapy were independent prognostic factors for OS and CSS in the ovarian group and for OS in the endometrial group. Lymph node metastasis and surgery were also independent prognostic factors for CSS in the endometrial group.

**Conclusion:**

Surgery alone may help to improve overall survival and CSS in patients with early-stage cervical LCNEC. In contrast, surgery+chemotherapy and surgery+radiotherapy may help to improve survival in those with early-stage ovarian and endometrial LCNEC, respectively. Regardless of subtype, comprehensive treatment involving surgery, CTX, and RT should be considered to improve prognosis in patients with advanced-stage gynecologic LCNEC.

## Introduction

Neuroendocrine tumors (NETs) are neuroendocrine cell-derived malignancies that can occur in various parts of the body, most commonly in the lungs. Among them, neuroendocrine carcinoma is a rare subtype that can be further classified into four types according to the College of American Pathologists and the National Cancer Institute: carcinoid, atypical carcinoid, large-cell carcinoma, and small-cell carcinoma. This classification is comparable to that used for NETs of the lung (1). In 2014, the World Health Organization (WHO) updated the classification of NETs occurring in different areas of the female genital tract, classifying them as either low-grade or high-grade NETs. Low-grade NETs include carcinoid and atypical carcinoid tumors, while high-grade NETs include small-cell carcinoma and large-cell carcinoma (LCC) (2–6).

The occurrence of gynecologic LCNEC is extremely rare, with most tumors arising in the cervix and following an aggressive clinical course (7, 8), followed by the ovary and endometrium. Previous studies have reported that cervical LCNEC accounts for only 0.087–0.6% of all cervical cancers (9, 10). At present, there are no survey-based data regarding the relative incidence of ovarian or endometrial LCNEC. However, data extracted from the Surveillance, Epidemiology, and End Results (SEER) database indicate that incidence rates for LCNEC of the ovary and endometrium are approximately 0.15% and 0.029%, respectively.

Given the low incidence of gynecologic LCNEC, most published articles are case reports or small case series. Such reports have highlighted the aggressive biological behavior and poor prognosis of gynecologic LCNEC, for which rates of recurrence and distant metastasis are high even in the early stages of disease. Gynecologic LCNEC has also been described as highly invasive and malignant, resulting in low survival rates (11–13). However, these previous studies examined single disease entities only, and cross-sectional analyses and comparisons remain lacking. Furthermore, treatments for gynecologic LCNEC are considered experimental. In principle, surgical treatments for cervical, ovarian, and endometrial LCNEC are the same as those for cervical squamous cell carcinoma, epithelial ovarian cancer, and endometrial adenocarcinoma, respectively. Furthermore, although the adjuvant chemotherapy scheme for gynecological LCNEC is similar to that for primary lung LCNEC, there is no consensus regarding the optimal treatment plan or prognostic factors for each site. In the present study, we aimed to address these issues by summarizing and comparing clinical characteristics, treatment methods, prognosis, and prognostic factors among cervical, ovarian, and endometrial LCNEC.

## Materials and methods

### Data source and patient selection

The SEER database of the National Cancer Institute, which covers 30 percent of the U.S. population across 14 states, provides cancer statistics including incidence and survival data for the targeted geographic areas. Using this database, we identified patients who had been histologically diagnosed with NETs from 1988 to 2015, selecting those with primary malignancies of the cervix, ovary, and uterine body (ICD-O-3/WHO 2008 website code; described as 8012/3: large-cell carcinoma and 8046/3: non-small-cell carcinoma). The exclusion criteria were as follows: previous benign or borderline tumor confirmed *via* autopsy or based on information from the patient’s death certificate, diagnosis of carcinoma in situ, not the first tumor, etc. SEER*Stat 8.3.9 software (https://seer.cancer.gov/data/) was used to generate the case list. Staging was determined in accordance with the American Joint Committee on Cancer (AJCC) staging system. As the SEER database is public and includes de-identified data only, approval from the local ethics committee was not required for the current analysis.

### Clinical and demographic characteristics

We analyzed demographic data including race (black, white, other, unknown), age at diagnosis (≥85 years, 65–84 years, 45–64 years, <45 years), marital status (widowed, divorced/separated, single/unmarried, married, unknown), insurance status (uninsured, requiring any medical assistance, insured, unknown), year of diagnosis (<2004, 2004–2009, 2010–2015), AJCC stage (I, II, III, IV, or unknown), grade (well/moderately/poorly differentiated, undifferentiated, unknown), lymph node status (not examined, positive, negative, unknown), and site of metastasis (bone, brain, liver, and lung [yes/no for each]). Data were also analyzed in terms of the following treatment patterns: surgery alone, surgery plus chemotherapy (surgery+CTX), surgery plus concurrent chemoradiotherapy (surgery+CCRT), surgery plus radiotherapy (surgery+RT), CTX alone, CCRT, RT alone, and no treatment.

### Statistical analysis

Clinical and demographic characteristics were compared among sites of gynecologic LCNEC using chi-square tests. Categorical data are presented as numbers and percentages, while quantitative data are presented as the means ± standard deviations. Univariate and multivariate Cox risk regression analyses were performed to identify independent predictors of overall survival (OS) and cancer-specific survival. OS durations were calculated using Kaplan–Meier plots and compared using the log-rank test. All data were analyzed using SPSS 25.0 software (SPSS, Chicago, IL, USA). Kaplan–Meier survival curves were drawn using GraphPad Prism (9.2.0 GraphPad Software, San Diego, CA, USA), and P values < 0.05 were considered statistically significant.

## Results

### Patients

A total of 467 women with gynecologic LCNEC registered in the SEER database fulfilled the criteria and were included in our study (**Table 1**), including 169 (36.2%), 219 (46.9%), and 79 (16.9%) with cervical, ovarian, and endometrial LCNEC, respectively. The median ages in the cervical, ovarian, and endometrial groups were 48.48 ± 15.24, 69.79 ± 13.54, and 55.37 ± 13.39 years, respectively. Patient characteristics, including AJCC stage, sampled pelvic nodes, grade, age, lymph node status, year of diagnosis, rates of distant metastasis, and treatment strategies, are summarized in **Table 1**.

**Table 1:** Demographic and clinical characteristics of patients with gynecologic large-cell neuroendocrine carcinoma (LCNEC).

Patient	Cervical LCNEC	Ovarian LCNEC	Endometrial LCNEC	
characteristics	N(%)	N (%)	N (%)	*P-value*
Mean age (years,SD)	48.48 (±15.24)	69.79 (±13.54)	65.37 (±13.39)	
ALL (467)	169 (36.2)	219 (46.9)	79 (16.9)	
**Age at diagnosis (years)**				**<0.001**
<45	81 (47.9)	8 (3.7)	5 (6.3)	
45-64	54 (32.0)	61 (27.9)	33 (41.8)	
65-84	32 (18.9)	120 (54.8)	35 (44.4)	
≥85	2 (1.2)	30 (13.6)	6 (7.5)	
**Race**				0.028
White	128 (75.8)	181 (82.6)	60 (75.9)	
Black	28 (16.5)	15 (6.9)	9 (11.3)	
Other	12 (7.2)	23 (10.5)	10 (12.8)	
Unknown	1 (0.5)	0 (0)	0 (0)	
**Marital status**				**<0.001**
Single/unmarried	36 (21.3)	22 (10.02)	15 (19.0)	
Married	74 (43.8)	92 (42.0)	31 (39.2)	
Divorced/separated	27 (16.0)	23 (10.5)	11 (14.0)	
Widowed	21 (12.4)	77 (35.1)	18 (22.8)	
Unknown	11 (6.5)	5 (2.2)	4 (5.0)	
**Insurance status**				
Insured	30 (17.7)	59 (26.9)	35 (44.3)	0.021
Any Medicaid	15 (8.8)	7 (3.2)	10 (12.6)	
Uninsured	4 (2.5)	1 (0.5)	3 (3.8)	
Unknown	120 (71.0)	152 (69.4)	31 (39.3)	
**AJCC Stage**				0.001
I	14 (8.3)	5 (2.3)	8 (10.1)	
II	8 (4.7)	5 (2.3)	5 (6.3)	
III	10 (5.9)	36 (16.4)	10 (12.7)	
IV	34 (20.2)	77 (35.2)	38 (48.2)	
Unknown	103 (60.9)	96 (43.8)	18 (22.7)	
**Year of diagnosis**				
<2004	103 (60.9)	83 (37.9)	18 (22.8)	**<0.001**
2004-2009	26 (15.4)	97 (44.3)	27 (34.2)	
2010-2015	40 (23.7)	39 (17.8)	34 (43.0)	
**Grade**				
Well differentiated	0 (0)	0 (0)	0 (0)	0.11
Moderately differentiated	1 (0.6)	0 (0)	0 (0)	
Poorly differentiated	18 (10.7)	54 (24.7)	34 (43.0)	
Undifferentiated	4 (2.7)	39 (17.8)	18 (22.8)	
Unknown	146 (86.4)	126 (57.5)	27 (34.2)	
**Lymph nodes status**				
Negative	24 (14.2)	17 (7.8)	15 (19.0)	0.002
Positive	16 (9.5)	21 (9.7)	5 (6.3)	
No examined	67 (39.6)	161 (73.5)	55 (69.7)	
Unknown	62 (36.7)	20 (9.0)	4 (5.0)	
**Sampled pelvic nodes**				0.02
1–9	13 (7.7)	18 (8.4)	7 (8.8)	
10–19	10 (5.9)	9 (4.1)	6 (7.6)	
≥20	16 (9.5)	11 (5.0)	7 (8.8)	
Not examined	63 (37.3)	161 (73.5)	55 (69.7)	
Unknown	67 (39.6)	20 (9.0)	4 (5.1)	
**Surgery performed**				0.01
Surgery	83 (49.1)	77 (35.2)	38 (48.1)	
No surgery	84 (49.7)	141 (64.3)	41 (51.9)	
Unknown	2 (1.2)	1 (0.5)	0 (0)	
**Chemotherapy**				
Yes	71 (42)	120 (57.5)	36 (45.5)	0.037
No	98 (58)	99 (45.2)	43 (54.4)	
**Radiotherapy**				
Yes	39 (23)	7 (3.1)	11 (13.9)	**<0.001**
No	130 (77)	212 (96.9)	68 (86.1)	
**Distant metastasis**				
bone	9 (5.3)	3 (1.4)	5 (6.3)	0.034
brain	3 (1.8)	0 (0)	2 (2.5)	
liver	6 (3.6)	10 (4.6)	7 (8.9)	
lung	12 (7.1)	4 (1.8)	10 (12.7)	
No	10 (5.9)	21 (9.6)	9 (11.4)	
Unknown	129 (76.3)	181 (82.6)	46 (58.2)	
**Treatment**				**<0.001**
Surgery alone	32 (18.9)	19 (8.7)	14 (17.8)	
Surgery + CTX	15 (8.9)	52 (23.8)	13 (16.5)	
Surgery + CCRT	17 (10.1)	6 (2.7)	6 (7.6)	
Surgery + RT	19 (11.2)	0 (0)	5 (6.3)	
CTX alone	36 (21.3)	62 (28.3)	17 (21.5)	
CCRT	3 (1.8)	0 (0)	0 (0)	
RT alone	0 (0)	1 (0.5)	0 (0)	
No treatment	47 (27.8)	79 (36.0)	24 (30.4)	

LCNEC, large-cell neuroendocrine carcinoma; RT, radiotherapy; CTX, chemotherapy; CCRT, concurrent chemoradiotherapy; AJCC, American Joint Commission on Cancer; black bold means p<0.05.

Age at onset was the highest in the ovarian group (69.79 years) and lowest in the cervical group (48.48 years), and significant differences in the prevalence of gynecologic LCNEC were observed among different age groups (P < 0.001). Most cases of cervical LCNEC occurred in patients <45 years old, while most cases of ovarian and endometrial LCNEC occurred in those who were 65–84 years old (54.8% and 44.4%, respectively). Most patients in each group were white (75.8% vs. 82.6% vs. 75.9%) (P = 0.028), and roughly 40% in each group were married (43.8% vs. 42.0% vs. 39.2%) (P < 0.001). Advanced-stage (stages III-IV) gynecologic LCNEC was more common in the endometrial group (60.9%) than in the cervical and ovarian groups (26.1% and 51.7%, respectively). Cervical LCNEC was primarily diagnosed before 2004 (60.9%), while ovarian and endometrial LCNEC were commonly diagnosed from 2004–2009 (44.3%) and from 2010–2015 (43.0%), respectively. The rate of lymph node dissection was higher in the endometrial group (25.3%) than in the cervical (23.7%) and ovarian (17.5%) groups. In terms of treatment strategies, CTX was more common in the ovarian group (54.8%) than in the cervical and endometrial groups (42.0% and 45.5%, respectively), while RT was more common in the cervical group (23.0%) than in the other two groups (ovarian: 3.1%; endometrial: 13.9%). Distant metastasis was common in patients with endometrial LCNEC (endometrial 30.4% vs cervical: 17.8%; ovarian: 7.8%).

### Survival curves

**Figure 1:**
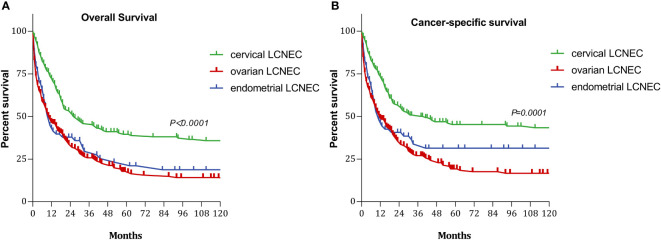
Survival curves with cervical, ovarian and endometrial LCNEC: **(A)** overall survival (OS); **(B)** cancer-specifi;c survival (CSS).

The 5-year OS rates for patients with cervical, ovarian, and endometrial LCNEC were 35.98%, 17.84%, and 23.21%, respectively, and the median duration of OS was 26, 11, and 11 months in each group. The 5-year CSS rates for the three groups were 45.23%, 19.23%, and 31.39%, respectively, and the median duration of CSS was 41, 12, and 11 months in each group (**Figure 1**).

**Figure 2:**
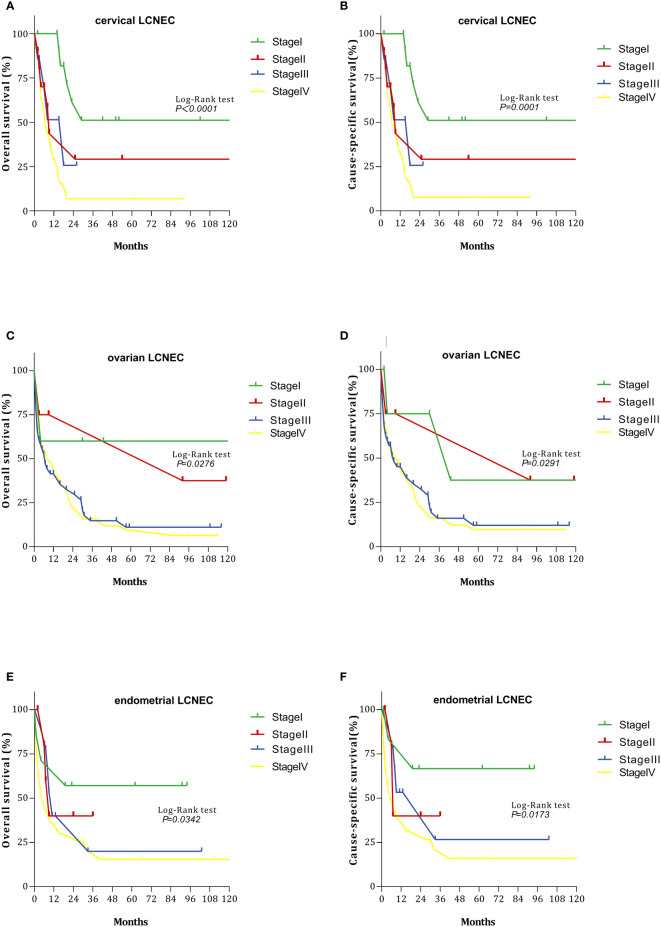
Survival curves with cervical, ovarian, and endometrial LCNEC at each stage: **(A)** overall survival (OS)with cervical LCNEC; **(B)** cancer-specific survival (CSS)with cervical LCNEC. **(C)** overall survival (OS)with ovarian LCNEC; **(D)** cancer-specific survival (CSS)with ovarian LCNEC; **(E)** overall survival (OS)with endometrial LCNEC; **(F)** cancer-specific survival (CSS)with endometrial LCNEC.

We also evaluated OS and CSS curves for various stages of gynecologic LCNEC (**Figure 2**). For cervical LCNEC, the 5-year OS rates for patients with stage I, II, III, and IV disease were 51.14%, 29.17%, 25.71%, and 6.81%, respectively; for ovarian LCNEC, they were 60.00%, 37.5%, 11.10%, and 6.36%, respectively; for endometrial LCNEC, they were 57.14%, 40.00%, 20.00%, and 15.41%, respectively. The 5-year CSS rates for patients with stage I, II, III, and IV cervical LCNEC were 51.14%, 29.17%, 25.71%, and 7.65%, respectively; for ovarian LCNEC they were 37.50%, 37.50%, 12.08%, and 9.72%, respectively; for endometrial LCNEC they were 66.67%, 40.00%, 26.67%, and 16.05%, respectively.

**Figure 3:**
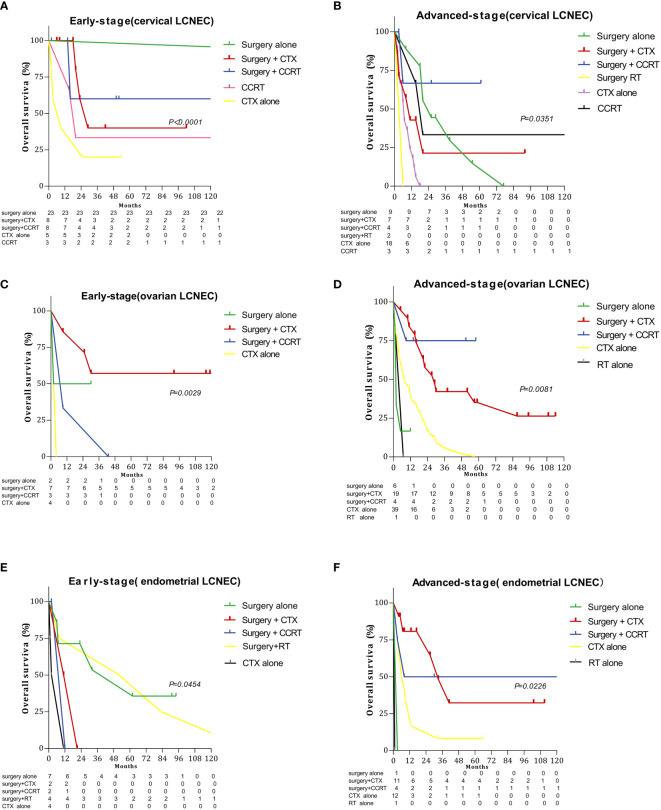
Survival curves for gynecologic LCNEC patients with early- and advanced-stage disease for different treatment regimens: **(A)** overall survival (OS) in the early stage with cervical LCNEC; **(B)** overall survival (OS) in the advanced stage with cervical LCNEC; **(C)** overall survival (OS) in the early stage with ovarian LCNEC; **(D)** overall survival (OS) in the advanced stage with ovarian LCNEC; **(E)** overall survival (OS) in the early stage with endometrial LCNEC; **(F)** overall survival (OS) in the advanced stage with endometrial LCNEC.

**Figure 4:**
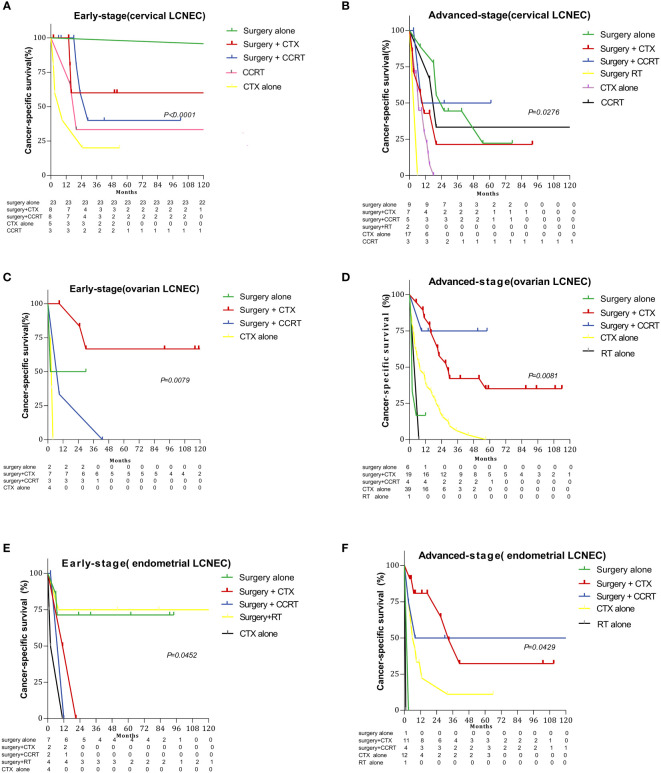
Survival curves for gynecologic LCNEC patients with early- and advanced-stage disease for different treatment regimens: **(A)** cancer-specific survival (CSS) in the early stage with cervical LCNEC; **(B)** cancer-specific survival (CSS) in the advanced stage with cervical LCNEC; **(C)** cancer-specific survival (CSS) in the early stage with ovarian LCNEC; **(D)** cancer-specific survival (CSS) in the advanced stage with ovarian LCNEC; **(E)**cancer-specific survival (CSS) in the early stage with endometrial LCNEC; **(F)** cancer-specific survival (CSS) in the advanced stage with endometrial LCNEC.

**Figures 3** and **4** summarize differences in prognosis based on treatment modality for patients with early- and advanced-stage LCNEC. Among patients with early-stage cervical LCNEC, the 5-year OS and CSS rates for cases treated with surgery alone were 95.65% and 95.65%, while those for patients with advanced cervical LCNEC treated with surgery+CCRT were highest at 66.67% and 50.00%, respectively. Among patients with early-stage ovarian LCNEC, the 5-year OS and CSS rates for cases treated with surgery+CTX were highest at 57.14% and 66.67%, while those for patients with advanced ovarian LCNEC treated with surgery+CCRT were highest at 75.00% and 75.00%, respectively. Among patients with early-stage endometrial LCNEC, the 5-year OS and CSS rates for cases treated with surgery+RT were 48.14% and 71.43%, respectively, while those for advanced cases treated with surgery+CCRT were 50.00% and 50.00%. These rates were also higher than those for other treatment options (**Table 2**).

**Table 2:** Univariate-Prognostic factors for gynecologic large-cell neuroendocrine carcinoma (LCNEC).

Subject	Overall survival						Cancer-specific survival					
Characteristics	Cervical LCNEC		Ovarian LCNEC		Endometrial LCNEC		Cervical LCNEC		Ovarian LCNEC		Endometrial LCNEC	
	HR (95%CI)	*P*-value	HR (95%CI)	*P-*value	HR (95%CI)	*P*-value	HR (95%CI)	*P*-value	HR (95%CI)	*P*-value	HR (95%CI)	*P*-value
**Age**												
<45	1	**<0.001**	1	**<0.001**	1	**0.01**	1	**0.001**	1	**<0.001**	1	**0.01**
45-64	3.068 (1.993-4.721)	<0.001	1.002 (0.453-2.215)	0.996	0.551 (0.189-1.606)	0.005	2.515 (1.573-4.020)	<0.001	0.95 (0.429-2.104)	0.899	0.511 (0.174-1,506)	0.003
65-84	3.019 (1.846-4.937)	<0.001	1.298 (0.604-2.791)	0.504	0.685 (0.238-1.969)	0.012	2.068 (1.177-3.633)	0.012	1.126 (0.552-2.428)	0.762	0.568 (0.194-1.664)	0.013
≥85	3.929 (0.942-16.393)	0.06	3.061 (1.326-7.063)	0.009	0.631 (0.164-2.419)	0.006	1.864 (0.254-13.663)	0.54	2.819 (1.219-6.518)	0.015	0.826 (0.22-3.099)	0.007
**AJCC stsge**												
I	1	**0.001**	1	0.532	1	0.188	1	**0.002**	1	0.331	1	0.078
II	1.976 (0.529-7.382)	0.311	0.557 (0.161-1.932)	0.357	1.848 (0.490-6.976)	0.365	1.98 (0.530-7.400)	0.310	0.44 (0.099-1.954)	0.280	2.208 (0.441-11.05)	0.335
III	4.252 (1.281-14.121)	0.018	1.217 (0.655-2.259)	0.534	1.220 (0.369-4.026)	0.745	4.349 (1.309-14.450)	0.016	1.266 (0.654-2.449)	0.484	1.617 (0.384-6.804)	0.512
IV	6.158 (2.351-16.130)	<0.001	1.230 (0.701-2.157)	0.470	2.403 (0.925-6.246)	0.072	5.723 (2.168-15.105)	<0.001	1.381 (0.760-2.510)	0.289	3.672 (1.11-12.152)	**0.03**
Unknown	NA		NA		NA		NA		NA		NA	
**Lymph nodes status**												
Negative	1	**0.002**	1	**<0.001**	1	**0**	1	**0.003**	1	**<0.001**	1	**0.01**
Positive	2.319 (0.932-5.773)	0.071	3.474 (1.418-8.512)	0.006	4.942 (1.209-20.209)	0.026	2.387 (0.908-6.277)	0.078	5.259 (1.738-15.911)	0.003	5.172 (1.149-23.289)	0.032
No examined	3.667 (1.743-7.715)	0.001	6.347 (2.935-13.727)	<0.001	7.058 (2.504-19.897)	<0.001	3.848 (1.741-8.505)	0.001	9.607 (3.523-26.198)	<0.001	6.666 (2.046-21.719)	0.002
Unknown	NA		NA		NA		NA		NA		NA	
**Surgery performed**												
Surgery	1		1		1		1		1		1	
No surgery	2.857 (1.954-4.178)	**<0.001**	3.565 (2.588-4.910)	**<0.001**	3.839 (2.178-6.675)	**<0.001**	2.807 (1.823-4.322)	**<0.001**	3.555 (2.554-4.948)	**<0.001**	3.611 (1.974-6.605)	**<0.001**
Unknown	NA		NA		NA		NA		NA		NA	
**Chemotherapy**												
Yes	1		1		1		1		1		1	
No	1.623 (1.107-2.379)	**0.013**	3.575 (2.625-4.869)	**<0.001**	1.402 (0.832-2.354)	**0.02**	1.513 (1.101-2.332)	**0.003**	3.419 (2.490-4.694)	**<0.001**	1.179 (0.674-2.062)	**0.03**
**Radiotherapy**												
Yes	1		1		**1**		1		1		1	
No	1.362 (0.87-2.133)	0.177	2.197 (0.815-5.921)	0.1199	1.647 (0.777-3.491)	0.193	1.297 (0.790-2.129)	0.305	2.082 (0.772-5.615)	0.148	1.998 (0.792-5.046)	0.143
**Distant metastasis**												
Yes	1		1.000		1.000		1		1.000		1	
No	0.187 (0.075-0.466)	**<0.001**	0.885 (0.399-1.961)	0.763	0.681 (0.282-1.644)	0.393	0.239 (0.103-0.555)	**0.001**	0.421 (0.454-2.298	0.960	0.58 (0.224-1.502)	0.262
Unknown	NA		NA		NA		NA		NA		NA	

AJCC, American Joint Commission on Cancer; LCNEC, large-cell neuroendocrine carcinoma; HR, hazard ratio; CI, confidence interval; NA, Not available;black bold means p<0.05.

**Figure 5:**
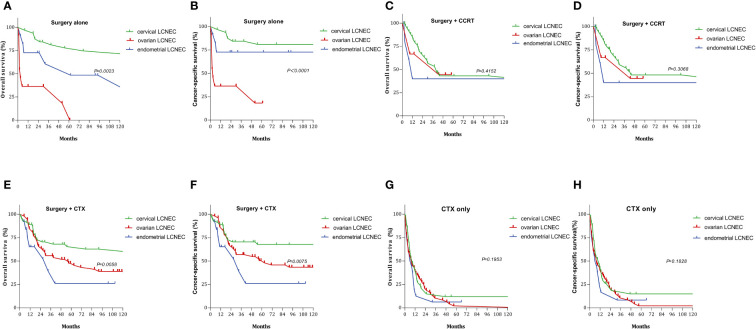
Survival curves for gynecologic LCNEC patients with different treatments: **(A)** overall survival (OS) in surgery alone with gynecologic LCNEC; **(B** cancer-specific survival (CSS) in surgery alone with gynecologic LCNEC; **(C)** overall survival (OS) in survery+CCRT with gynecologic LCNEC; **(D)** cancer-specific survival (CSS) in survery+CCRT with gynecologic LCNEC; **(E)** overall survival (OS) in surgery+CTX with gynecologic LCNEC **(F)** cancer-specific survival (CSS) in surgery+CTX with gynecologic LCNEC; **(G)** overall survival (OS) in CTX only with gynecologic LCNEC **(H)** cancer-specific survival (CSS) in CTX only with gynecologic LCNEC.

We also examined the effect of different treatments on prognosis in the cervical, ovarian, and endometrial groups. Among patients treated with surgery alone, the 5-year OS rates were 77.89%, 18.18%, and 60.61%, while the 5-year CSS rates were 81.00%, 18.18%, and 72.73%, respectively. Among patients treated with surgery+CTX, the 5-year OS rates were 65.44%, 47.64%, and 26.11%, while the 5-year CSS rates were 66.77%, 50.42%, and 26.11%, respectively. Among patients treated with surgery+CCRT, the 5-year OS rates 43.14%, 44.44%, and 40.00%, while the 5-year CSS rates were 48.23%, 44.44%, and 40.00%, respectively. The 5-year OS rates for patients treated with CTX only were 11.93%, 2.09%, and 6.25%, respectively, while the 5-year CSS rates were 14.91%, 2.13%, and 8.33%, respectively (**Figure 5**). Among patients treated with surgery alone, 5-year OS and CSS rates were highest in the cervical group and lowest in the ovarian group (OS: P=0.0023, CSS: P < 0.0001). Among patients treated with surgery+CTX, these rates were also best in the cervical group, although they were worst in the endometrial group (OS: P = 0.0058, CSS: P = 0075). There were no significant differences in OS or CSS rates among the three LCNEC sites for patients treated CTX only or surgery+CCRT (CTX only: OS: P = 0.195, CSS: P = 0.182; surgery+CCRT: OS: P = 0.415, CSS: P = 0.306).

### Prognostic factors

Univariate and multivariate analyses of age, AJCC stage, lymph node status, surgery, CTX, RT, and distant metastasis were used to identify prognostic factors for gynecologic LCNEC (**Tables 2**, **3**). Multivariate analysis revealed that lymph node metastasis, chemotherapy, and AJCC stage were independent prognostic factors for OS and CSS in patients with cervical LCNEC. Lymph node metastasis, chemotherapy, and surgery were independent prognostic factors for OS and CSS in the ovarian group and for OS in the endometrial group. Lymph node metastasis and surgery were also independent prognostic factors for CSS in the endometrial group (**Table 3**).

**Table 3:** Multivariate-Prognostic factors for gynecologic large-cell neuroendocrine carcinoma (LCNEC).

Subject		Overall survival					Cancer-specific survival					
Characteristics	Cervical LCNEC		Ovarian LCNEC		Endometrial LCNEC		Cervical LCNEC		Ovarian LCNEC		Endometrial LCNEC	
	HR (95%CI)	*P*-value	HR (95%CI)	*P*-value	HR (95%CI)	*P-*value	HR (95%CI)	*P*-value	HR (95%CI)	*P-*value	HR (95%CI)	*P*-value
**Age**												
<45	1	0.948	1	0.598	1	0.441	1	0.517	1	0.459		0.314
45-64	0.945 (0.309-2.892)	0.922	0.71 (0.313-1.608)	0.411	0.411 (0.134-1.258)	0.119	0.908 (0.290-2.843)	0.868	0.686 (0.301-1.563)	0.37	0.338 (0.108-1.056)	0.062
65-84	0.798 (0.200-3.186)	0.749	0.756 (0.343-1.665)	0.488	0.463 (0.156-1.373)	0.165	0.368 (0.063-2.151)	0.267	0.659 (0.298-1.457)	0.303	0.385 (0.127-1.169)	0.092
≥85			0.959 (0.397-2.318)	0.927	0.347 (0.073-1.652)	0.184			0.896 (0.369-2.177)	0.808	0.447 (0.095-2.091)	0.306
**AJCC stsge**												
I	1	**0.010**	–	–	–	–	1	**0.01**	–	–	–	–
II	6.799 (0.232-28.884	0.266	–	–	–	–	8.208 (0.304-21.489)	0.211	–	–	–	–
III	10.898 (5.802-48.739)	0.003	–	–	–	–	12.820 (9.807-25.969)	0.002	–	–	–	–
IV	17.619 (5.125-68.660)	0.006	–	–	–	–	15.503 (5.120-52.590)	0.006	–	–	–	–
Unknown	NA		NA				NA					
**Lymph nodes status**												
Negative	1	**0.003**		**0.004**		**0.008**		**0**		**0.003**		**0.028**
Positive	1.177 (0.115-12.071)	0.891	2.425 (0.967-6.081)	0.059	9.321 (2.079-41.797)	0.004	0 (0-0.054)	0.002	3.607 (1.173-11.093)	0.025	8.432 (1.709-41.602)	0.009
No examined	0.001 (0.001-0.059)	0.001	3.748 (1.646-8.534)	0.002	4.845 (1.528-15.358)	0.007	0.896 (0.080-10.046)	0.929	5.502 (1.935-15.650)	0.001	4.348 (1.149-16.457)	0.03
Unknown	NA		NA		NA		NA		NA		NA	
**Surgery performed**												
Surgery	1		1		1		1		1		1	
No surgery	6.4 (0.487-84.182)	0.158	2.157 (1.406-3.310)	**<0.001**	2.672 (1.336-5.347)	**0.005**	0.132 (0.009-1.961)	0.141	2.158 (1.389-3.350)	**0.001**	2.659 (1.229-5.754)	**0.013**
Unknown	NA		NA		NA		NA		NA		NA	
**Chemotherapy**												
Yes	1		1		1		1		1		1	
No	20.2 (3.848-106.034)	**<0.001**	3.165 (2.207-4.539)	**<0.001**	1.898 (1.053-3.422)	**0.033**	46.514 (5.804-372.76)	**<0.001**	2.984 (2.058-4.327)	**<0.001**	1.476 (0.780-2.974)	0.232
**Distant metastasis**												
Yes	1		–	–	–	–	1		–	–	–	–
No	0.806 (0.236-2.758)	0.731	–	–	–	–	0.888 (0.254-3.11)	0.853	–	–	–	–
Unknown	NA						NA					

AJCC, American Joint Commission on Cancer; LCNEC, large-cell neuroendocrine; HR, hazard ratio; CI, confidence interval; NA, Not available; black bold means p<0.05.

## Discussion

Given our limited understanding regarding the occurrence, development, and pathogenesis of gynecologic LCNEC, we examined the clinical behavior of the disease *via* a retrospective analysis of data from 469 patients, representing the largest cohort of patients with gynecologic LCNEC in the literature to date. No previous studies have performed such comparisons among different subtypes of gynecologic LCNEC, highlighting the practical significance of the current results for guiding clinical work.

Our data suggest that gynecologic LCNEC exhibits a unique natural history and aggressive clinical course, with the highest and lowest survival rates occurring in patients with cervical and ovarian disease, respectively. The results of our analysis suggest that surgery alone can improve OS and CSS in patients with early-stage LCNEC (I/II). In contrast, surgery+CTX and surgery+RT may help improve survival for early-stage ovarian and endometrial LCNEC, respectively. Moreover, regardless of subtype, our data suggest that comprehensive treatment with surgery, CTX, and RT should be considered to improve prognosis in patients with advanced-stage gynecologic LCNEC.

Embry et al. (11) reported 62 cases of cervical LCNEC, representing the largest series thus far, in which the median patient age was 37 years, and the median duration of OS was 16.5 months (0.5–151 months). In their multivariate analysis, early-stage disease and CTX treatment were associated with improved survival. The use of platinum agents and platinum plus etoposide treatment were also associated with improved survival. Nonetheless, recurrence was observed in 70% (38/54) of patients, and 40% (25/62) of cases were classified as stage IV. In their study of 45 patients with cervical LCNEC, Burkeen et al. (12) reported a median age of 36 years and a median OS duration of 16 months, with early cases (I/II) accounting for 73%. Lee and Ji (13) reported a case of cervical LCNEC treated with radical surgery and CCRT. After a disease-free period of 18 months, the patient experienced three consecutive recurrences in the kidney, breast, and adrenal gland, respectively, and survived for a total of 63 months. Habeeb and Habeeb (14) reported a case in which a patient with stage IIA2 disease died 21 months postoperatively despite treatment with CTX and palliative RT. In the current study, the median age of patients with cervical LCNEC was 48.48 (n=169). The median durations of OS and CSS in the cervical group were 26 and 41 months, respectively, which are longer than those reported in the two largest studies mentioned above. However, the OS and CSS rates for cervical LCNEC were still only 35.98% and 45.23% at 5 years.

The principles of surgical treatment for cervical LCNEC are the same as those used for cervical squamous cell carcinoma. Strategies involving postoperative adjuvant systemic CTX combined with RT have been developed primarily based on data from patients with LCNEC of the lung (1, 6, 7, 11, 12). Our results suggest that surgery alone can improve OS and CSS for early-stage cervical LCNEC, while surgery+CCRT can improve survival for advanced-stage LCNEC, highlighting the need for a targeted treatment approach. Some somatostatin receptor binding is commonly observed in patients with high-grade NETs. Therefore, Shahabi et al. (15) suggested exploring targeted therapy with octreotide, a somatostatin analog, while Kajiwara et al. proposed a somatostatin type 2A analog for the treatment of tumor cells expressing somatostatin type 2A receptors (16). However, this strategy has not been standardized and requires further study.

The metastatic epidemiology of cervical LCNEC remains unclear, as only a few relevant case reports have been published (17, 18). Given the aggressive nature of the disease, early metastases to the peripheral lymph nodes, lung, liver, bone, and brain have been reported (19, 20). Our study included 12 cases of lung metastasis, nine of bone metastasis, six of liver metastasis, and three of brain metastasis. Treatment data in cases of recurrence are limited in the SEER database. Tempfer et al. (21) demonstrated the potential value of immune checkpoint inhibitors, while other studies have noted that nivolumab and the MEK inhibitor trametinib can be considered (22, 23). In their study, Carroll et al. (24) demonstrated that pure high-grade neuroendocrine cervical cancer is microsatellite stable, with most patients exhibiting negative PD-L1 expression. Since most of the tumors tested expressed PARP-1, future clinical trials may wish to include PARP inhibitors for patients with recurrent high-grade neuroendocrine cervical cancer.

The largest case series for ovarian LCNEC included 58 patients, although only 15 cases were classified as pure ovarian LCNEC, and the median survival time was only 10 months. These results emphasize that even patients with stage I disease are likely to experience a very poor prognosis (25). In their study of 45 patients with ovarian LCNEC, Burkeen et al. reported that the majority of patients had advanced stage (III/IV) disease (12). Among the 33 cases reported by Oshita et al., the 5-year OS rate was only 34.9% (26). Lin et al. also reported a case in which a patient with stage IV primary pure ovarian LCNEC with liver metastases was treated with three cycles of postoperative paclitaxel + carboplatin, noting that she experienced disease progression with pulmonary metastases and died 3 months after surgery (27). Ki et al. reported three cases of stage I LCNEC characterized by poor survival due to biological invasiveness, despite extensive surgery and CTX (28). Among the 219 cases of ovarian LCNEC in our study, median OS and CSS durations were 11 and 12 months, and the 5-year OS and CSS rates were only 17.84% and 19.23%, respectively. Among these patients, 113 had advanced disease, accounting for 51.7% of cases. The 5-year OS and CSS rates were lower for ovarian LCNEC than for cervical or endometrial LCNEC, and liver metastasis was noted in 10 cases of ovarian LCNEC. These results highlight the need to examine factors that place patients at high risk for poor prognosis following a diagnosis of ovarian LCNEC, as well as those associated with prognosis.

A previous study reported that the overexpression of synaptophysin is an independent contributor to poor prognosis, based on a multivariate analysis that included age, FIGO stage, and postoperative residual tumors (29). Our multivariate results indicated that lymph node metastasis, surgery, and CTX are independent prognostic factors for OS and CSS in patients with ovarian LCNEC, emphasizing the need to focus on surgery and postoperative adjuvant CTX in these patients. In principle, surgery for ovarian LCNEC is equivalent to that for epithelial ovarian cancer. Our research shows that surgery+CTX should be recommended for early-stage ovarian LCNEC, while surgery+CCRT should be recommended for advanced cases. As the SEER database does not include data regarding sites of recurrence for ovarian LCNEC, further studies are required to clarify this issue and identify effective treatments for recurrent ovarian LCNEC.

Endometrial LCNEC is a rare malignancy that appears to exhibit an aggressive course even in early stages, with a strong tendency for distant metastasis and rapid recurrence (6, 7, 30). Over 19 years, 18 Japanese medical institutions have accumulated only 14 cases of endometrial LCNEC, including seven each of the mixed and pure types (31), highlighting the extremely low incidence of the disease. The prognosis appears to be significantly worse for pure cases than that for mixed cases but significantly better for cases treated with surgery than without and for those in which surgery is incomplete. Radical surgery should therefore be considered for endometrial LCNEC. Tu et al. (32) reported that cytoreductive surgery was suboptimal in a patient with stage IV disease. Despite receiving platinum-based adjuvant chemotherapy after surgery, the patient developed obstructive ileus developed 2 months later, and he died 8 days after ileus surgery. Suh et al. (33) reported a case in which stage IIIB endometrial LCNEC demonstrated a progressive course even after surgery, multiple postoperative CTX and RT regimens (etoposide-cisplatin, irinotecan-cisplatin), and FOLFIRI (fluorouracil, leucovorin, irinotecan) treatment. In their case, lymph node metastasis was identified 12 months after surgery, and the patient died 23 months after surgery. Nguyen et al. (30) reported a stage IVB endometrial LCNEC in a 71-year-old patient who underwent surgical debulking, who survived for only 32 days after surgery. Both studies described the disease as exhibiting a rapidly progressive course.

Among the 79 patients with endometrial LCNEC in our study, 48 had advanced-stage disease, accounting for 60.9% of cases, and the median age at onset was 55.37 years. In these patients, the median OS and CSS durations were both 11 months, and the 5-year OS and CSS rates were 23.21% and 31.39%, respectively. Among patients with endometrial LCNEC, we observed 10 cases of lung metastasis, seven of liver metastasis, five of bone metastasis, and two of brain metastasis. These results suggest that endometrial LCNEC often occurs in the advanced stage, highlighting its strong invasiveness and risk of poor prognosis. Standard surgical procedures for endometrial cancer include total hysterectomy, bilateral salpingo-oophorectomy, and lymph node dissection, although omentectomy is performed in the absence of endometrioid histology. Similar surgical procedures have been used in most patients with endometrial LCNEC. However, given its low incidence and the apparent risk of metastasis, it seems that a multimodal treatment approach should be utilized for endometrial LCNEC. Our results suggest that surgery+RT can improve OS and CSS in patients with early-stage endometrial LCNEC, while surgery+CCRT should be considered for advanced cases. In addition to histologic subtype, only complete surgery was an important prognostic factor in our multivariate analysis. In accordance with this finding, Matsumoto et al. (7) also reported that complete surgery can improve the prognosis of early to advanced endometrial LCNEC. Our multivariate analysis for endometrial LCNEC indicated that lymph node metastasis, surgery, and CTX were independent prognostic factors for OS, while lymph node metastasis and surgery were independent prognostic factors for CSS.

Adjuvant chemotherapy options for gynecologic LCNEC are similar to those for primary pulmonary LCNEC, including platinum-based chemotherapy and paclitaxel-carboplatin-based chemotherapy (34). Several strategies have been employed, including cisplatin+cyclophosphamide, etoposide+cisplatin, paclitaxel+carboplatin (12, 35). Cisplatin+vinorelbine and other regimens have been used for tumors that have failed to respond to first-line therapy (12). Irinotecanplatin or topotecan can be considered as second-line therapy for gynecologic LCNEC (34–37), while octreotide, a synthetic somatostatin analog, represents a therapeutic option for combination CTX (16).

Establishing the diagnosis of gynecologic LCNEC can be challenging. LCNECs are characterized by the presence of large polygonal cells as well as a low nucleocytoplasmic ratio and thick nuclear chromatin, with prominent peripheral palisades and frequent glandular differentiation. Gynecologic LCNEC must therefore be assessed *via* immunohistochemical analysis, as these tumors exhibit a positive immune response to at least one neuroendocrine marker such as synaptophysin, chromogranin A, neuron-specific alkene-positive immunostaining for alcoholase or CD56, or p63 (38). Such immune responses are therefore used to confirm the diagnosis.

Our study had some limitations. Because LCNEC is high-grade by definition, no variable analyses were performed for grade. In addition, although the SEER database separates reports of simple LCNEC and mixed LCNEC, we performed analyses for simple LCNEC only, which is less common than mixed LCNEC. Furthermore, specific information on CTX, RT, and disease recurrence is not included in the SEER database, highlighting the need to accumulate data from additional cases to guide future clinical work. Because the incidence rate of cervical LCNEC is higher than that of ovarian or endometrial LCNEC, a relatively higher number of cervical LCNEC cases were included in the current study, and methods for surgical intervention and postoperative adjuvant treatment are more standardized for cervical LCNEC than for the other two disease entities. Indeed, data are largely lacking for recurrent ovarian and endometrial LCNEC at present. This limitation underscores the importance of accumulating additional cases to aid in the development of targeted treatment strategies for cases for ovarian and endometrial LCNEC, such as immunotherapy or gene detection. Such data may in turn help to improve survival among patients with rarer forms of LCNEC.

## Conclusion

The current study, which represents the largest analysis of gynecologic LCNEC thus far, demonstrates that surgery should be used for initial treatment. Surgery+CTX or RT can be used in early-stage cases, while both CTX and RT should be used for advanced cases. Additional studies involving larger numbers of cases are required to determine the most appropriate strategies for treating these aggressive tumors. Establishing a global database of gynecologic LCNEC may aid in designing retrospective and prospective studies of such strategies. Furthermore, future studies may wish to focus on the molecular and genetic aspects of targeting NETs to improve survival in patients with gynecologic LCNEC.

## Data availability statement

The original contributions presented in the study are included in the article/supplementary material. Further inquiries can be directed to the corresponding author.

## Author contributions

LP: clinical data collection, conceptualization, data curation, formal analysis, and writing - original draft. JC: clinical data collection and conceptualization. XC: data curation and writing - review and editing. All authors significantly contributed to the study and approved the submitted version.

## Conflict of interest

The authors declare that the research was conducted in the absence of any commercial or financial relationships that could be construed as a potential conflict of interest.

## Publisher’s note

All claims expressed in this article are solely those of the authors and do not necessarily represent those of their affiliated organizations, or those of the publisher, the editors and the reviewers. Any product that may be evaluated in this article, or claim that may be made by its manufacturer, is not guaranteed or endorsed by the publisher.

## References

[1] Travis WD Linnoila RI Tsokos MG Hitchcock CL Cutler GB Nieman L. Neuroendocrine tumors of the lung with proposed criteria for large-cell neuroendocrine carcinoma. an ultrastructural, immunohistochemical, and flow cytometric study of 35 cases. Am J Surg Pathol (1991) 15:529–53. doi: 10.1097/00000478-199106000-00003 1709558

[2] Walenkamp AM Sonke GS Sleijfer DT. Clinical and therapeutic aspects of extrapulmonary small cell carcinoma. Cancer Treat Rev (2009) 35:228–36. doi: 10.1016/j.ctrv.2008.10.007 19068273

[3] Brennan SM Gregory DL Stillie A Herschtal A Mac Manus M Ball DL. Should extrapulmonary small cell cancer be managed like small cell lung cancer? Cancer (2010) 116:888–95. doi: 10.1002/cncr.24858 20052730

[4] van Meerbeeck JP Fennell DA De Ruysscher DK. Small-cell lung cancer. Lancet (2011) 378:1741–55. doi: 10.1016/S0140-6736(11)60165-7 21565397

[5] Frazier SR Kaplan PA Loy TS. The pathology of extrapulmonary small cell carcinoma. Semin Oncol (2007) 34:30–8. doi: 10.1053/j.seminoncol.2006.11.017 17270663

[6] Winer I Kim C Gehrig P. Neuroendocrine tumors of the gynecologic tract update. Gynecol Oncol (2021) 162:210–19. doi: 10.1016/j.ygyno.2021.04.039 34023130

[7] Gardner GJ Reidy-Lagunes D Gehrig PA. Neuroendocrine tumors of the gynecologic tract: A society of gynecologic oncology (SGO) clinical document. Gynecol Oncol (2011) 122:190–8. doi: 10.1016/j.ygyno.2011.04.011 21621706

[8] Crowder S Tuller E. Small cell carcinoma of the female genital tract. Semin Oncol (2007) 34:57–63. doi: 10.1053/j.seminoncol.2006.10.028 17270667

[9] Wang KL Wang TY Huang YC Lai JC Chang TC Yen MS. Human papillomavirus type and clinical manifestation in seven cases of large-cell neuroendocrine cervical carcinoma. J Formos Med Assoc (2009) 108:428–32. doi: 10.1016/S0929-6646(09)60088-7 19443298

[10] Albores-Saavedra J Gersell D Gilks CB Henson DE Lindberg G Santiago H. Terminology of endocrine tumors of the uterine cervix: Results of a workshop sponsored by the college of American pathologists and the national cancer institute. Arch Pathol Lab Med (1997) 121:34–9. 9111090

[11] Embry JR Kelly MG Post MD Spillman MA. Large Cell neuroendocrine carcinoma of the cervix: Prognostic factors and survival advantage with platinum chemotherapy. Gynecol Oncol (2011) 120:444–8. doi: 10.1016/j.ygyno.2010.11.007 21138780

[12] Burkeen G Chauhan A Agrawal R Raiker R Kolesar J Anthony L. Gynecologic large cell neuroendocrine carcinoma: A review. Rare Tumors (2020) 12:2036361320968401. doi: 10.1177/2036361320968401 33194158 PMC7605029

[13] Lee E Ji YI. Large Cell neuroendocrine carcinoma of the cervix with sequential metastasis to different sites: A case report. Case Rep Oncol (2018) 11:665–70. doi: 10.1159/000493912 PMC6243902 30483095

[14] Habeeb A Habeeb H. Large Cell neuroendocrine carcinoma of the uterine cervix. BMJ Case Rep (2019) 12:bcr–2018. doi: 10.1136/bcr-2018-225880 PMC6340556 30642849

[15] Shahabi S Pellicciotta I Hou J Graceffa S Huang GS Samuelson RN. Clinical utility of chromogranin a and octreotide in large cell neuro endocrine carcinoma of the uterine corpus. Rare Tumors (2011) 3:e41. doi: 10.4081/rt.2011.e41 22355496 PMC3282446

[16] Kajiwara H Hirabayashi K Miyazawa M Nakamura N Hirasawa T Muramatsu T. Immunohistochemical expression of somatostatin type 2A receptor in neuroendocrine carcinoma of uterine cervix. Arch Gynecol Obstet (2009) 279:521–5. doi: 10.1007/s00404-008-0760-y 18726606

[17] Hooker N Mohanan S Burks RT. A rare presentation of stage IV large cell neuroendocrine carcinoma of the cervix with metastasis to the cranium. Case Rep Obstet Gynecol (2018) 2018:2812306. doi: 10.1155/2018/2812306 30013803 PMC6022268

[18] Ono K Yokota NR Yoshioka E Noguchi A Washimi K Kawachi K. Metastatic large cell neuroendocrine carcinoma of the lung arising from the uterus: A pitfall in lung cancer diagnosis. Pathol Res Pract (2016) 212:654–7. doi: 10.1016/j.prp.2016.03.009 27113439

[19] Nagao S Miwa M Maeda N Kogiku A Yamamoto K Morimoto A. Clinical features of neuroendocrine carcinoma of the uterine cervix: A single-institution retrospective review. Int J Gynecol Cancer (2015) 25:1300–5. doi: 10.1097/IGC.0000000000000495 26166556

[20] Sato Y Shimamoto T Amada S Asada Y Hayashi T. Large Cell neuroendocrine carcinoma of the uterine cervix: A clinicopathological study of six cases. Int J Gynecol Pathol (2003) 22:226–30. doi: 10.1097/01.PGP.0000071046.12278.D1 12819387

[21] Tempfer CB Tischoff I Dogan A Hilal Z Schultheis B Kern P. Neuroendocrine carcinoma of the cervix: A systematic review of the literature. BMC Cancer (2018) 18:530. doi: 10.1186/s12885-018-4447-x 29728073 PMC5935948

[22] Sharabi A Kim SS Kato S Sanders PD Patel SP Sanghvi P. Exceptional response to nivolumab and stereotactic body radiation therapy (SBRT) in neuroendocrine cervical carcinoma with high tumor mutational burden: Management considerations from the center for personalized cancer therapy at UC San Diego moores cancer center. Oncologist (2017) 22:631–7. doi: 10.1634/theoncologist.2016-0517 PMC5469598 28550027

[23] Lyons YA Frumovitz M Soliman PT. Response to MEK inhibitor in small cell neuroendocrine carcinoma of the cervix with a KRAS mutation. Gynecol Oncol Rep (2014) 10:28–9. doi: 10.1016/j.gore.2014.09.003 PMC4434143 26075998

[24] Carroll MR Ramalingam P Salvo G Fujimoto J Solis Soto LM Phoolcharoen N. Evaluation of PARP and PDL-1 as potential therapeutic targets for women with high-grade neuroendocrine carcinomas of the cervix. Int J Gynecol Cancer (2020) 30:1303–7. doi: 10.1136/ijgc-2020-001649 PMC8375494 32727929

[25] Yang X Chen J Dong R. Pathological features, clinical presentations and prognostic factors of ovarian large cell neuroendocrine carcinoma: A case report and review of published literature. J Ovarian Res (2019) 12:69. doi: 10.1186/s13048-019-0543-z 31345245 PMC6657379

[26] Oshita T Yamazaki T Akimoto Y Tanimoto H Nagai N Mitao M. Clinical features of ovarian large-cell neuroendocrine carcinoma: Four case reports and review of the literature. Exp Ther Med (2011) 2:1083–90. doi: 10.3892/etm.2011.325 PMC3440829 22977625

[27] Lin CH Lin YC Yu MH Su HY. Primary pure large cell neuroendocrine carcinoma of the ovary. Taiwan J Obstet Gynecol (2014) 53:413–6. doi: 10.1016/j.tjog.2013.06.017 25286804

[28] Ki EY Park JS Lee KH Bae SN Hur SY. Large Cell neuroendocrine carcinoma of the ovary: A case report and a brief review of the literature. World J Surg Oncol (2014) 12:314. doi: 10.1186/1477-7819-12-314 25314924 PMC4210534

[29] Shakuntala PN Uma Devi K Shobha K Bafna UD Geetashree M. Pure large cell neuroendocrine carcinoma of ovary: A rare clinical entity and review of literature. Case Rep Oncol Med (2012) 2012:120727. doi: 10.1155/2012/120727 23304586 PMC3523574

[30] Nguyen ML Han L Minors AM Bentley-Hibbert S Pradhan TS Pua TL. Rare large cell neuroendocrine tumor of the endometrium: A case report and review of the literature. Int J Surg Case Rep (2013) 4:651–5. doi: 10.1016/j.ijscr.2013.04.027 PMC3710906 23792474

[31] Matsumoto H Shimokawa M Nasu K Shikama A Shiozaki T Futagami M. Clinicopathologic features, treatment, prognosis and prognostic factors of neuroendocrine carcinoma of the endometrium: A retrospective analysis of 42 cases from the Kansai clinical oncology Group/Intergroup study in Japan. J Gynecol Oncol (2019) 30:e103. doi: 10.3802/jgo.2019.30.e103 31576694 PMC6779616

[32] Tu YA Chen YL Lin MC Chen CA Cheng WF. Large Cell neuroendocrine carcinoma of the endometrium: A case report and literature review. Taiwan J Obstet Gynecol (2018) 57:144–9. doi: 10.1016/j.tjog.2017.12.025 29458887

[33] Suh DS Kwon BS Hwang SY Lee NK Choi KU Song YJ. Large Cell neuroendocrine carcinoma arising from uterine endometrium with rapidly progressive course: Report of a case and review of literature. Int J Clin Exp Pathol (2019) 12:1412–7. PMC6947078 31933957

[34] Matsumoto H Nasu K Kai K Nishida M Narahara H Nishida H. Combined large-cell neuroendocrine carcinoma and endometrioid adenocarcinoma of the endometrium: A case report and survey of related literature. J Obstet Gynaecol Res (2016) 42(2):206–10. doi: 10.1111/jog.12881 26807962

[35] Shepherd FA Evans WK MacCormick R Feld R Yau JC. Cyclophosphamide, doxorubicin, and vincristine in etoposide- and cisplatin-resistant small cell lung cancer. Cancer Treat Rep (1987) 71:941–4. 2820571

[36] Makihara N Maeda T Nishimura M Deguchi M Sonoyama A Nakabayashi K. Large Cell neuroendocrine carcinoma originating from the uterine endometrium: A report on magnetic resonance features of 2 cases with very rare and aggressive tumor. Rare Tumors (2012) 4:e37. doi: 10.4081/rt.2012.e37 23087793 PMC3475944

[37] O’Brien ME Ciuleanu TE Tsekov H Shparyk Y Cuceviá B Juhasz G. Phase III trial comparing supportive care alone with supportive care with oral topotecan in patients with relapsed small-cell lung cancer. J Clin Oncol (2006) 24:5441–7. doi: 10.1200/JCO.2006.06.5821 17135646

[38] Pocrnich CE Ramalingam P Euscher ED Malpica A. Neuroendocrine carcinoma of the endometrium: A clinicopathologic study of 25 cases. Am J Surg Pathol (2016) 40:577–86. doi: 10.1097/PAS.0000000000000633 PMC5998806 26945341

